# Representations of electronic cigarettes in Chinese media

**DOI:** 10.1186/s12889-018-5644-x

**Published:** 2018-06-13

**Authors:** Shaojing Sun, Giuseppe A. Veltri, Fan Wang

**Affiliations:** 1 School of Journalism, Fudan University, Shanghai, China; 2 Department of Sociology and Social Research, University of Trento, Trento, Italy; 3 Key Laboratory of Public Health Safety, Ministry of Education, School of Public Health, Fudan University, Shanghai, 200032 P. R. China

**Keywords:** E-cigarettes, Tobacco promotion, Chinese media, Text-mining approach, Tobacco control

## Abstract

**Background:**

Electronic cigarettes (E-cigarettes) have become a debated issue for tobacco control over recent years. In this study we investigate how Chinese newspapers have covered E-cigarettes over the past ten years.

**Methods:**

The study analyses the salience, patterns and content of news articles pertaining to E-cigarettes in regional and national Chinese outlets. A total of 476 articles are examined via content analysis and supervised automatic text analysis. The manual content analysis generates a coding scheme, which is then validated and applied to machine learning. The whole research methodology demonstrates satisfying human-human and human-to-computer reliabilities.

**Results:**

The study reveals that E-cigarettes have not received enough attention in terms of its salience in the media, though the amount of coverage has been growing. A large share of the articles is published around May of each year – which is when the No Tobacco Day of the WHO takes place. The results point to four major themes on E-cigarettes: nicotine/constituents/features, tobacco control/regulation, children’s use of E-cigarettes, and tobacco market/industry.

**Conclusions:**

Overall, E-cigarettes have not been a topic at the top of media agenda; however, we have identified a considerable growth of coverage about the potential concerns regarding young people’s adoption of E-cigarettes advocated by parents and educators.

## Background

Electronic cigarettes (E-cigarettes) have become a debated issue for tobacco control over recent years. According to the WHO, E-cigarettes are defined as electronic devices that do not burn or use tobacco leaves but rather vaporize a solution that consumers inhale (see the link) [1]. On one hand, the products are increasingly accepted by smokers and nonsmokers; on the other hand, there is a lack of definitive evidence on their potential health effects [2].

Grana, Benowitz, and Glantz reviewed existing studies and noted a lack of evidence supporting E-cigarettes as an effective tool of reducing harm in comparison to smoking combustible cigarettes [3]. Moreover, an increasing amount of research is pointing to a high possibility of dual use of E-cigarettes and conventional cigarettes, as well as a growing rate of youth initiation with E-cigarettes. In other words, E-cigarette can serve as a gateway to consuming combustible cigarettes by youth [4].

Considering the ongoing controversy on E-cigarettes, the present study explores Chinese newspaper coverage of E-cigarettes over the past ten years. Such an endeavor is valuable, as media (e.g., newspapers) serve as an important source for the general public to acquire information about E-cigarettes [5]. Examining media coverage of E-cigarettes may provide insight into public responses to and governmental regulations of the products, too [6].

### Tobacco control and E-cigarettes in China

Tobacco control has long been a challenging task in China, a country housing more than 1.3 billion people in the world. It is reported that about 52.4% of adult men and 3.4% of adult women smoke [7]. Furthermore, it is estimated that smoke-related diseases will eventually kill more than 150 million current smokers in China [8]. Despite the formidable threat of tobacco consumption, progress in tobacco control is far from satisfying in this country. For instance, in 2006, China signed into the Framework Convention on Tobacco Control (FCTC). And yet, to date, no national law has been established to target tobacco control in public settings across China.

With the emergence of E-cigarettes, the situation for tobacco control in China has only become more complex and challenging. According to the most recent report by Chinese CDC in 2015, about 27.7% of Chinese people aged 15 and above smoke, amounting to a total of 316 million current smokers. About 40.5% of those surveyed people have heard about E-cigarettes, and about 3.1% of them had experience of using E-cigarettes [9]. Furthermore, to our knowledge, Chinese government has not issued any law or public regulation on E-cigarettes.

Pepper and Brewer pointed out several issues about the regulation of E-cigarettes including safety information, public interest, and scientific research [10]. For instance, researchers observed that safety and cessation are the major themes appearing in marketing or advertising E-cigarettes, and many of those claims (e.g., healthier alternative or harmless) are unsubstantiated or overstated [11]. On the other hand, Li, Xiao, Chu, Qin, and Wang, surveying 1188 current smokers in Beijing, reported that over 2/3 of the smokers adopted E-cigarettes for the purpose of quitting conventional smoking [12].

### Media communication of E-cigarettes

As mentioned earlier, up to now, there is no definitive conclusion on the long-term health effects of consuming E-cigarettes [3]. As such, complete approval or denial of the products may be premature. The increasing popularity and adoption of E-cigarettes, at least, is partly due to the prevalence of information about the products and their usage on various media platforms. For example, news coverage of tobacco-related issues has grown significantly in newspapers in China over the past years [13]. Meanwhile, messages about E-cigarettes diffused exponentially to a more diverse audience due to the expanding social media network [14].

In addition to the growing amount of communication, media messages about E-cigarettes also seem to be chaotic and even misleading. Recently, Rooke and Amos content analyzed 119 articles from UK and Scottish newspapers published from 2007 to 2012, and identified five major themes about E-cigarettes [15]. The themes are related to smoke-free legislation, risk and uncertainty about health effects, being healthier choice compared to traditional cigarettes, celebrity use, and price. Among these themes, ‘healthier choice’ is the one which appears in media articles most frequently. Concretely, E-cigarettes are presented as a healthier alternative to smoking conventional cigarettes or suggested to be less harmful. A comprehensive analysis of American newspapers also showed that media have presented a mixed view, both positive and negative, of E-cigarettes [5]. Over half of the articles emphasized the need for more knowledge about the health effects of consuming E-cigarettes.

### Media agenda-setting

The way media cover E-cigarettes could potentially influence what people think about and how they think about the products. Furthermore, it is argued that media such as newspapers are powerful to influence policy-makers’ decision making due to their in-depth and complete coverage of issues [16]. Such media impacts are termed agenda-setting in media communication [17]. Specifically, the first level agenda-setting characterizes the general amount of media coverage on a certain object or issue, namely, the salience of an issue in the news. Intense media coverage can render an issue salient enough that people will pay more attention to it. In contrast, second level agenda-setting speaks to the features of media content and the attributes of an object or issue.

A concept central to agenda-setting theory is media salience, which is multidimensional in nature [18]. More concretely, media salience is comprised of the dimensions including attention, prominence, and valence. Attention is typically gauged by the volume of media stories on a particular issue. Prominence is characterized by factors such as page placement, size of headlines, and the prestige of media covering the issue. Valence often features the overall tone (e.g., positive, negative, neutral) of the coverage or the amount of conflict in media stories. According to Kiousis, the dimensions of attention and prominence reflect the visibility of a topic, whereas the valence dimension reflects the internal qualities of a topic or object covered by media [18].

Departing from the media agenda-setting theory, the present study is aimed to investigate how Chinese newspapers have been covering E-cigarettes over the past years. Furthermore, we are interested in exploring the amount, themes, angles, and changes of newspaper coverage. Such an exploration, hopefully, will help shed light on consuming and regulating E-cigarettes, particularly in developing countries such as China.

## Methods

### Sample of articles

To start, we searched the Wisers database (http://www.wisers.com), the largest and authoritative database of Chinese newspapers. The database is updated every day with articles from over 12,000 media sources. A total of 1050 different Chinese newspapers were located in the database. In the database, Chinese newspapers are classified into two types: national newspapers circulating across the country and regional newspapers circulating in local cities. From a different perspective, the newspapers can be categorized into comprehensive type (e.g., *People’s Daily*) and specialized type (e.g., *Health News*). We referenced prior research articles [15, 19], along with consulting experts in tobacco control and media communication, to compile a list of keywords relating to E-cigarettes such as electronic cigarettes, vaping, vape, ENDS, among others. These English keywords, plus their Chinese translations, were utilized to search for relevant articles.

**Fig. 1:**
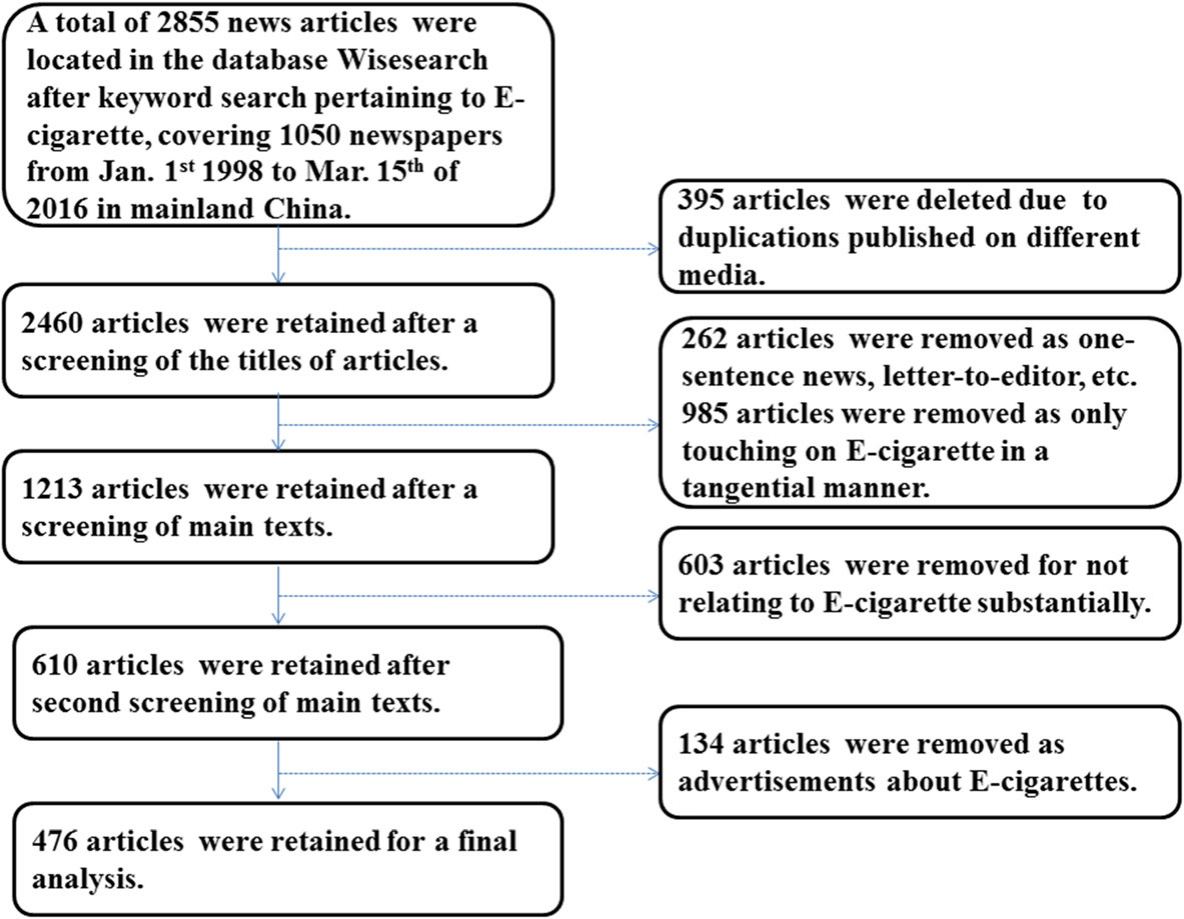
Procedure of Screening Articles. The arrows depict the flow process of how we screen articles across stages

A general search with the keywords generated 2855 news articles. After deleting duplicates, advertisements, letters to editor, as well as those not substantially related to E-cigarettes, 476 articles were retained for data analysis. The dates of the retained articles spanned from March of 2004 to March of 2016. A detailed description of the screening procedure is presented in Fig. 1.

### Analytical procedure

#### Coding and classification scheme

We adopted a multistep analytic induction method for individual and joint analysis of the news articles [20]. First, we coded the descriptive characteristics of each article and newspaper, including newspaper type (national vs. regional, comprehensive vs. specialized), publication time (year and month), article type (e.g., editorial and news), article length, and page placement (front-page or not).

Second, one of the authors read a sample of 200 articles line-by-line to identify “thought units,” in accordance with the definition of E-cigarettes and related topics thereof (e.g., activities, conflicts, stakeholders). A thought unit could be short or long, depending on whether it conveys complete meaning of thought [21]. For example, the following words are coded as one unit, “the journalist went to a store off campus and found different brands of E-cigarettes. Then the journalist came to the office store on campus and found the E-cigarettes which interviewed students smoked. The manager explained that, the products came to the shelf starting last year, and more students are coming to buy the products this year.” Although this passage is long, the three sentences, on the whole, convey one essential thought that students are the buyers of E-cigarettes.

After training with data from a different project on tobacco control, a graduate assistant unitized the same 200 articles from the present study and reached an intercoder reliability of .95 (Krippendorff’s alpha) with the author. The assistant and the author then proceeded to finish unitizing all the 476 articles. The unitizing process generated a total of 2591 units.

The third step involves merging units and creating higher-order categories or themes. Two of the authors with expertise in E-cigarettes followed the constant comparative method to examine, refine, merge, and reexamine categories [20]. The authors consulted with past research [5] to help identify valid themes. The coding and refining process was also guided by a few general questions (e.g., what characteristics of E-cigarettes are stressed? What are the uses of E-cigarettes presented for? Who are the main actors in the article?) [15]. When two coders cannot agree with a theme, a third researcher with expertise in tobacco control and content analysis was brought into the discussion for a decision. In the extreme case, a majority vote was adopted to settle the disagreement [22]. Taking into account of the exhaustiveness of the whole coding scheme and exclusiveness of individual categories, the two authors reached consensus on the final coding scheme. To safeguard against the possibility of obscuring any additional themes caused by our unitization and categorization, the authors went back to reexamine the articles with particular attention to the portion of articles which were not coded as units pertaining to E-cigarettes. The findings were further discussed between the authors and used to refine the coding scheme.

#### Supervised automatic text analysis

Another data analytic strategy we employed is automatic text analysis, with the unit of analysis as a meaningful word or phrase. After preprocessing data (e.g., stemming and removing stop words from the texts), we inspected those keywords that most frequently co-occur with the term E-cigarettes, so as to build a general context for understanding the topic. Such an automatic text analysis involves three steps including constructing training data, classifying articles, and validating results [23]. Two hundred articles were utilized for machine learning, in light of the earlier created coding scheme, to construct contexts for the themes related to E-cigarettes.

The automatic text analysis applied TDF-IDF (term frequency–inverse document frequency) normalisation and scaling to lexical units with presence/absence values. Then employing cosine coefficients as the measure, the analysis yielded a multidimensional scale (MDS map). The MDS map presents keywords represented on a two-dimensional scale in terms of their proximity reflecting their co-occurrences in the corpus. In this study, we introduce a more complex analysis applying hierarchical clustering based techniques to a non-Indo-European corpus for the first time. These analytical methods have been used before on corpora in Indo-European languages but not in Mandarin.

We compared the computer-coded results with the manually coded results, and the human-to-computer coding agreement reached 97%, ranging from 94 to 100% on the four themes. We then applied supervised machine learning to the rest of the corpus, and validated the classification accuracy of articles in accordance with the procedure detailed by Scharkow [24].

## Results

We analyzed 476 articles from more than one hundred newspapers, with 20.8% coming from national outlets (e.g., *People’s Daily*) and 79.2% from regional ones (e.g., *Wuhan Evening News*). Furthermore, 75.6% articles were from comprehensive newspapers whereas 24.4% were from specialized ones (e.g., *Health Times*). The lengths of articles ranged from 96 to 6079 Chinese words, with a median of 849 words. The results indicate that E-cigarettes appear to be a topic of limited national or political weight, as a majority of the articles were published in regional newspapers, which are typically less reputable than national ones. Very few articles were published on front pages. Moreover, even specialized newspapers focusing on health news have not given much attention to E-cigarettes, as we have not found any relevant articles in some health-focused newspapers (e.g., *Health Newspaper*).

**Fig. 2:**
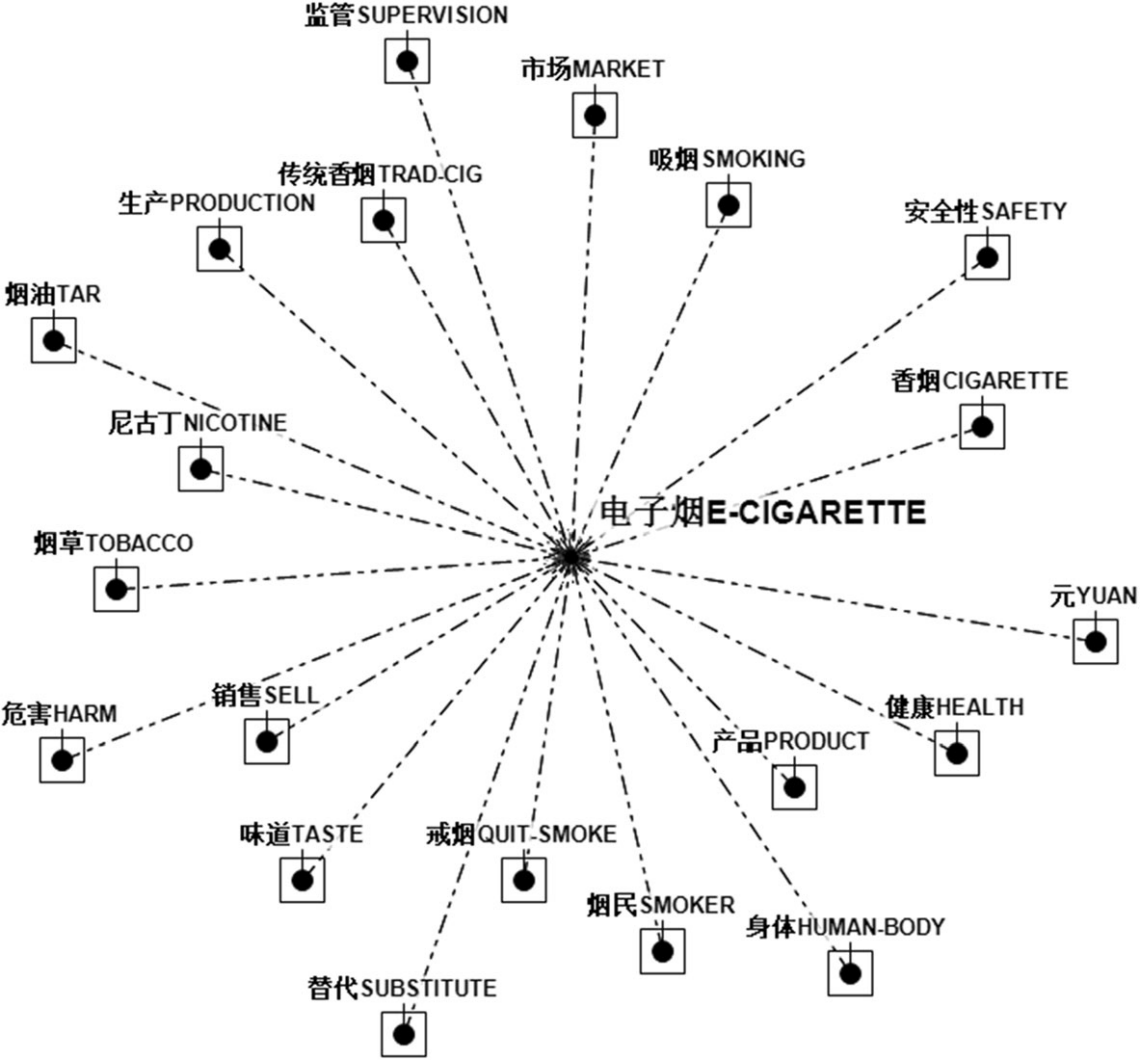
Word-Association Test on E-cigarettes. The dots denote different keywords. The lengths of the connection line denotes how closely two words are linked to each other. The shorter the length, the more closely two keywords are related to each other

**Fig. 3:**
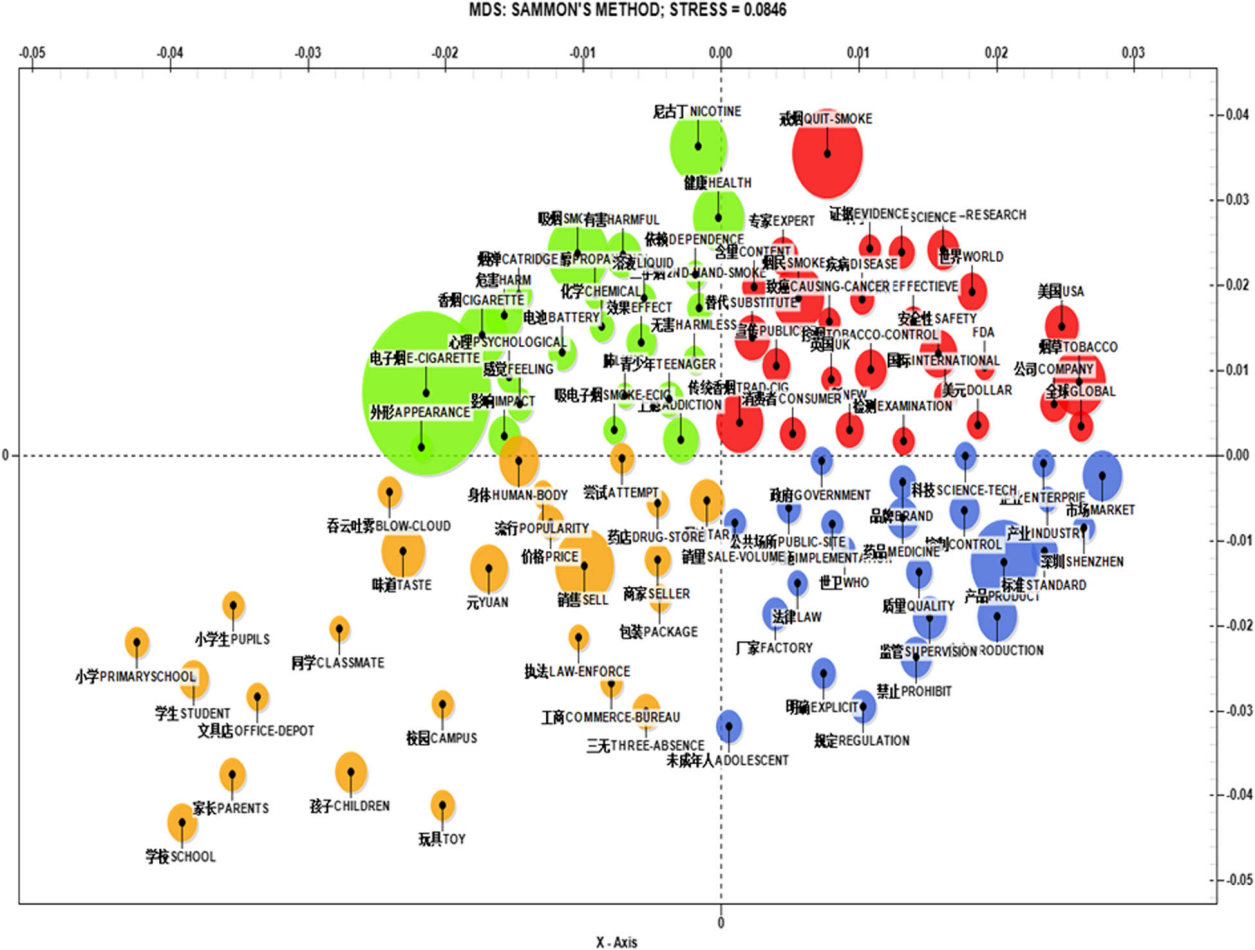
Multidimensional Scaling of Keywords. The size of each solid dot represents the weight a keyword contributes to a theme. The four different colors denote four different themes as described in the main text

Automatic text analysis (see Fig. 2) revealed that the keywords most closely associated with E-cigarettes are 戒烟 (smoking cessation) and产品 (product). In other words, E-cigarettes are frequently depicted in the context of smoking cessation and product marketing. Media often portrayed E-cigarettes as an aid for quitting conventional smoking. On the other hand, the industry of E-cigarettes has been thriving over recent years. Some terms (e.g., harm, safety) associated with E-cigarettes do convey a valence about the products. However, a majority of the associative keywords (e.g., product, market) seem to be neutral. A more detailed mapping of the contextual keywords is presented in Fig. 3.

The content analysis revealed four major themes about E-cigarettes in the newspapers. Presented in Table 1 are the details about the themes, with each comprised of 3 to 6 subcategories. Also presented are the percentage of articles referencing each category and the percentage of units constituting that category.

**Table 1:** Percentage of articles and total units coded regarding each category

E-cigarettes categories	# of articles referencing each category (% of articles)	# of units coded into each category (% of total units)
nicotine/constituents/features	418(88%)	749 (29%)
1. ingredients	203 (43%)	209 (8%)
2. flavor and experience	123 (26%)	131 (5%)
3. appearance and features of E-cigarettes	111 (23%)	115 (4%)
4. working mechanism of E-cigarettes	78 (16%)	82 (3%)
5. impact on health	123 (26%)	130 (5%)
6. functions of E-cigarettes	81 (17%)	82 (3%)
tobacco control/regulation	312 (66%)	493 (19%)
1. aids for smoking cessation	125 (26%)	135 (5%)
2. regulations on smoking E-cigarettes	103 (22%)	124 (5%)
3. science on smoking	76 (16%)	77 (3%)
4. smoking and campaign	64 (13%)	64 (2%)
5. tobacco control in general	85 (18%)	93 (4%)
children’s use of E-cigarettes	398 (84%)	830 (32%)
1. attractions of E-cigarettes to kids	123 (26%)	145 (6%)
2. use of E-cigarettes on campus	201 (42%)	225 (9%)
3. marketing nearby schools	199 (42%)	221 (9%)
4. parents’ and teachers’ concerns	212 (45%)	239 (9%)
tobacco market/industry	284 (60%)	519 (20%)
1. producers/brands/sales	219 (46%)	225 (9%)
2. quality and standards of products	202 (42%)	205 (8%)
3. trends of the industry	83 (17%)	89 (3%)
other	52 (11%)	16 (1%)

The first theme mainly describes the ingredients or components of E-cigarettes and hence is labelled as ‘nicotine/constituents/features’. The results showed that 88% of the articles mentioned this theme, which is the most prevalent in the news. Exemplar descriptive keywords include nicotine, tar, propanediol, cartridge, chemical, dependence, atomization, etc. One exemplar quote from local newspaper *Hai Xi Morning News* (Oct. 10, 2015) is as below:

“*headline: watch out! It is not credible that E-cigarettes are harmless.*

*… according to the city CDC, most cigarettes on market are cigarette-like devices comprised of lithium batteries, atomizer, and cartridge. Through the atomizer, the device converts Nicotine liquid into vapor, so that one can inhale in. So, E-cigarettes also contain nicotine which is addictive …*”.

In the news, E-cigarettes are often described by being *anchored* to conventional cigarettes. Terms such as nicotine and tar tell about the linkage between electronic and conventional products. On the other hand, E-cigarettes do consist of new components such as cartridge and battery. All these concrete attributes speak to the process of E-cigarettes objectification (i.e., turning something abstract into more concrete), a term from the social representations theory. This particular theme captures the potential effects of consuming E-cigarettes on individual health.

The second theme, is labelled as ‘children’s use of E-cigarettes.’ The results showed that 84% of the articles mentioned this theme, which is the second most prevalent in the news. Exemplar descriptive keywords include “campus,” “toy,” “popular,” “taste,” vaping,” “appearance,” “price,” “office-store,” “attempt,” among others. E-cigarettes are frequently sold nearby schools so that adolescents can have easy access to these products. Below is one exemplar quote from *Shao Yang Evening News* (Dec 12th, 2015):

“*headline: students vaping, parents worrying.*

*Mrs Wang told the reporter that when she passed by the fourth middle school, she saw several students smoking colorful cigarettes and vaping to show off in front of other students. As the parent, she was very worried about this.*”

Also, in the media, E-cigarettes are described with its taste, appearance, and personal experience of consuming them. Children enjoy the fruit-like taste and the coolness of vaping. Moreover, children are proud of consuming the product in their own social network settings. The coolness, colorfulness, appearance, popularity of the product make teenagers easily identify with it. One quote from the *Southern Metropolitan Newspaper* (April 10, 2015) put:

“*headline: the boss of convenience store said E-cigarettes are popular, regretting not to have entered the market earlier.*

*Some parents called the reporter that convenience stores nearby the campus are selling E-cigarettes, and some students come to buy them … One interviewed primary-school student said smoking E-cigarettes is very cool. Some female students even bought E-cigarettes as presents for males.*”

In this presentation of E-cigarettes, children tend to perceive them as toys, instead of tobacco products. It largely remains a vacuum in terms of the safety and effect of consuming E-cigarettes among teenagers. Due to absence of supervision and regulation, the products are publicly on shelf in toy-stores, office-stores, and convenience stores nearby schools.

The third theme, is labeled as tobacco control and regulation. The results showed that 66% of the articles mentioned this theme. This theme centers on the progress of tobacco control across the world. Specifically, recent public opinions or research findings from different sources (e.g., USA, UK, and WHO) are frequently quoted to illustrate the effect and regulation of E-cigarettes. Keywords associated with this theme include “science,” “research,” “WHO,” “U.S.A.,” “evidence,” etc. An article from a local newspaper, the *New Commerce Newspaper* (July 17, 2015) states:

“*headline: E-cigarettes not effective on quit-smoking, but also harmful to health.*

*…a recent study published on the American Public Health confirmed, E-cigarettes are not helpful for quitting smoking, but also may lead to addiction again. The WHO does not encourage people to use E-cigarettes as a tool to help quit smoking….*”

The fourth theme, is labeled as tobacco market/industry. The theme, mentioned in 60% of the news articles, is captured by the following keywords such as “production,” “standard,” “quality,” “market,” “brand,” “price,” and “Shen Zhen city.” Notably, Shen Zhen has been frequently mentioned as the major site for producing E-cigarettes in China. This theme features the production, marketing, and regulation of E-cigarettes. For instance, news articles have presented the products as cheap, innovative, and massive in production. The whole industry, however, has no established consistent standards, regulations, and supervision. An important keyword pertaining to this theme is “three No” (no standard for products, no supervision for quality, no evaluation for safety). The local newspaper *Wu Zhong Daily* (April 8, 2015) published the following:

“*don’t let E-cigarettes harm children!*

*… E-cigarettes on the market are not in the scope of supervision… the government has not classified E-cigarettes as tobacco products, which results the absence of supervision. … 国家烟草专卖局 (the State Tobacco Monopoly Administration) pointed out that E-cigarettes contain Nicotine, and hence should be monitored as tobacco products. Some experts believe E-cigarettes should be classified as medical devices as they have certain effects on quitting-smoke. So, E-cigarettes should be under the supervision of 食品药品监督局 (the Food and Drug Administration). Some people contended, there is no confirmation of E-cigarettes’ effect on quitting smoke. As such, they should be viewed as typical electronic devices, and should be supervised by 国家质量技术监督局 (the State Bureau of Quality and Technical Supervision)…*”.

**Fig. 4:**
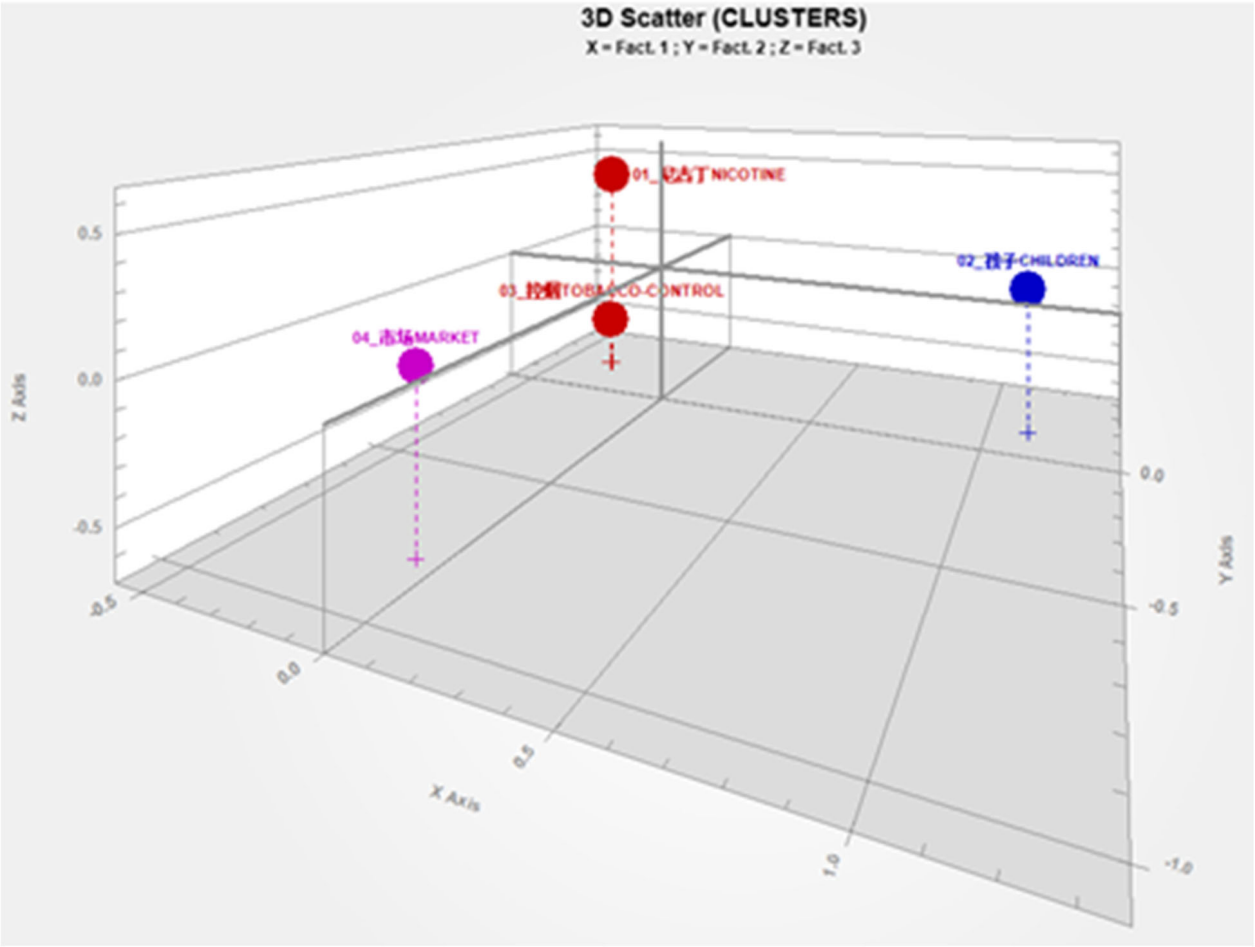
3-D Plot of Main Themes. The four solid dots denote four different themes, as labeled in the graph. If two dots fall into the same cubic region, that mean the two represented themes are tightly related to each other

The 3-D plot (Fig. 4) presents the relationships between the four themes. The two themes, nicotine/constituents and tobacco control, located in the same coordinate space, are more closely related to each other as compared to other themes.

Our results showed that the reporting of E-cigarettes spiked in the last two years. The earliest coverage on E-cigarettes dates back to 2004, attesting to the newness of the topic in China. The theme ‘children’s use of E-cigarettes’ has become pronounced in 2015 and attracted a substantial amount of coverage ever since. Also, there is a slight increase of coverage on the themes of nicotine/constituents bearing on the health effects of E-cigarettes. In contrast, media coverage of the theme of market/industry remains to be relatively stable. However, we did not find any significant difference of reporting E-cigarettes across media types (e.g., national vs. regional, comprehensive vs. specialized).

## Discussion

To our knowledge, this is the first study comprehensively analyzing Chinse newspaper coverage of E-cigarettes. Comparing the present study with prior ones will help situate our findings in a broader and more meaningful context.

Although E-cigarettes have received growing media attention over recent years, the amount of media coverage on the whole is imbalanced across time and media platforms. A large share of the articles came out around May of each year – which is when the No Tobacco day of the WHO takes place - and most articles were published in regional newspapers [13]. Our findings were, in general, consistent with prior studies. For instance, Liu et al. analyzed leading Chinese newspapers and found that the number of articles on conventional smoking peaked around May [25]. A plausible interpretation is that the “World No Tobacco Day” (WNTD) of May 31st attracts substantial media attention, driving media frenzy on tobacco issues during the time of May each year. Such a finding meshes with prior research, indicating that the WNTD has a significant impact on media coverage of and public interest in smoking cessation [26].

He, Shen, Yin, Xu, and Lan found significant differences between high-level Chinese party newspapers and local newspapers in covering tobacco control [27]. For instance, the former ones provided more coverage on tobacco industry, advertisement, and promotion than the latter, which focused more on education and smoking prevention. But, our study did not show any significant finding on comparing media types. The findings are more sensible from the perspective of Chinese media control and censorship. As Chan explained, Chinese government is highly concerned with how media influence public opinion and further social stability [28]. E-cigarettes, relating to tobacco control, could be a topic which the government exerts restraints on reporting so as to make the coverage across media align with each other.

Wallington, et al. found that both structural factors (e.g., the size of news organizations) and individual characteristics (e.g., journalists’ education level) are significant precursors of media agenda setting and framing [29]. Specifically, those factors can influence the sources, angles, and priorities that journalists employ in reporting health and medical science news. As mentioned earlier, about 80% articles were published in regional media outlets, which impacts are not comparable to those of national newspapers. Journalists affiliated with regional media may have more freedom to cover new topics, but that freedom also varies across different columns or sections of the newspapers [28]. As our data have shown, most of those articles on E-cigarettes were published on sections pertaining to social life, science/technology, or even international news. Furthermore, the lack of related articles on front pages has made the topic less salient to the public. As such, E-cigarettes have not been positioned high on the media agenda from the perspective of first-level agenda setting theory.

Our finding has lent support to the media salience argument bearing on agenda-setting theory. Specifically, E-cigarettes are a kind of marginalized topic in terms of the intensity, placement, and tone of coverage. For the present study, the analyzed articles are mostly informative, without showing a strong position or valence. This is also evidenced by the majority of neutral keywords associated with E-cigarettes. However, Yates et al. analyzed 450 American newspaper articles and found significant differences in reporting E-cigarettes between informative (e.g., news) and opinion pieces (e.g., editorials) [5]. The former type of articles focused more on explaining the new devices and controversy around its usage, whereas the latter one focused more on the potential benefits and popularity of the product.

Our study revealed a lack of attention paid to the topic of E-cigarettes by national media and health-focused newspapers. To our knowledge, it is pretty much a vacuum of regulating E-cigarettes in China. Both the general public and policy makers are not fully aware of the necessity and urgency to regulate E-cigarettes. As such, media do have an important role to play with respect to drawing attention to the issue. As a matter of fact, sales of E-cigarettes have brought decent revenues to the producers and marketers. Yet, in comparison, the general public possess very little information on the fast developing industry [30]. Better knowledge of the sales and marketing strategies is a prerequisite of enacting effective regulations on the products.

The present study revealed that about 1/3 of the news articles have voiced concerns about the attraction of E-cigarettes to teenagers. In those articles, school teachers and parents are the most frequently interviewed or quoted sources. Such reports are instances of a second-level agenda setting or framing in this context. E-cigarettes are represented as potential risks to teenagers. The main discourse associated with the risk in the newspapers is about the shining attractiveness of E-cigarettes to young consumers. As a recent study revealed, people adopted E-cigarettes out of various reasons such as social image and quitting combustibles [31]. The glamor and fashion surrounding E-cigarettes have constructed a new sub-culture, which strongly appeals to youth. Such an E-cigarette culture has been dubbed as “cloud chasing” (e.g., blowing smoke rings) [32]. Researchers also reported additional features (e.g., modifiable components of the device (mods), pairing of mods with juices) appealing to consumers [33].

### Implications for tobacco control

In general, the appeal of E-cigarettes to consumers, particularly young consumers, cannot be underestimated. For instance, Peters, Meshack, Lin, Hill, and Abughosh studied a sample of 47 teenage boys and found that a majority of these E-cigarette users deemed the products as readily accessible, healthier than conventional cigarettes, and aesthetically pleasing [34]. Nonnemaker, Kim, Lee, and MacMonegle surveyed American smokers and found that the most valued attributes of E-cigarettes were the perceived less harmful health effect and easiness to consume in public places [35]. Kong, Morean, Cavallo, Camenga, & Krishnan-Sarin, found that adolescents initiated the use of E-cigarette mainly out of the reasons for personal curiosity, product flavors, easy access, perceptions as cool or sexy, among others [36]. E-cigarettes have been marketed on the media as a more fashionable and safer alternative to conventional cigarettes.

Regulating such products and preventing children from accessing E-cigarettes could be the emerging agenda of tobacco control policies. Furthermore, as Doward and Agerholm reported, teenagers were driven to consuming E-cigarettes mainly by factors including personal choice, socialization, and peer group status [32]. Media have reported that there is a growing concern about teenagers consuming these products in school environments and peer gatherings. Interviewed young consumers often use words such as “coolness,” “novelty,” and “good taste” to describe their experience. Teasing out the renormalization process of consuming E-cigarettes would be conducive to regulating the new tobacco products.

Although a raft of media reports voiced the concern about children’s access to E-cigarettes, scientific evidence on the health effects of consumption is further needed. For a long time, implementation of tobacco control has been criticized for not being well connected with scientific advances on the health effects of tobacco consumption [25]. As such, journalists and media professional should follow and make better use of scientific evidence to advocate for regulating E-cigarettes. Doing so may lead to higher salience of the issue on both public and policy agenda, and hence advance the institution of corresponding laws and regulations in China.

### Limitations and future research directions

While this is the first study about e-cigarettes media coverage in China, we have analyzed only newspapers published in mainland China. As a result, our study cannot capture viewpoints from other communication outlets, particularly social media platforms. Indeed, the Internet has become a main avenue for the tobacco industry to market E-cigarettes [14]. The need to monitor and regulate information about E-cigarettes on new media cannot be understated for the benefit of public health. Furthermore, those marketing messages through retweet network can quickly reach certain potentially vulnerable populations such as young adults. As a recent study by Chu et al. demonstrated, the messages from an E-cigarette brand (i.e., Blu) diffused rapidly beyond the E-cigarette community to a much wider audience through the Twitter network [14]. We envisage the goal of extending our study in the future to include social media sources is highly scalable to larger data collection. Future research also calls for using social media data and potentially combining several sources in a ‘big data’ approach.

Regulating the consumption of E-cigarettes is a complex, scientific, cultural and social issue [5]. Future studies should look into the various emerging tobacco products in different nations and cultures. Digging into the societal and cultural factors will inform tobacco control in general and E-cigarette regulation in particular.

## Conclusions

Our study showed that although Chinese media coverage has been increasing, the topic of E-cigarettes is not salient on the media overall. We found that children and students are the major population of concern for consuming E-cigarettes in Chinese media. We also noticed that the media call for the regulation of marketing and consuming E-cigarettes is on the rise in China. We consider the current study as a step forward in analyzing and mapping out the landscape of reporting E-cigarettes in China which will allow cross-cultural comparisons and the extension to other media platforms.

## Abbreviations

E-cigarettes: Electronic cigarettes
LSA: latent semantic analysis
WHO: World Health Organization
